# Association of two types of dietary pattern scores with cardiovascular disease risk factors and serum 25 hydroxy vitamin D levels in Saudi Arabia

**DOI:** 10.29219/fnr.v65.5481

**Published:** 2021-06-02

**Authors:** Najlaa M. Aljefree, Noha M. Almoraie, Israa M. Shatwan

**Affiliations:** Food and Nutrition Department, Faculty of Human Sciences and Design, King Abdulaziz University, Jeddah, Saudi Arabia

**Keywords:** vitamin D deficiency, diet score, high-fat dietary pattern, Mediterranean diet, cardiovascular disease, obesity, low-density lipoprotein

## Abstract

**Background:**

Cardiovascular disease (CVD) is a main cause of mortality and disability worldwide. One of the key factors in the soaring prevalence of CVD globally has been nutrition transitions and changes in dietary patterns.

**Objective:**

This study investigated the association between two diet scores, namely, a high-fat dietary (HFD) pattern score and a Mediterranean diet (MedDiet) score, and CVD risk factors (obesity, hypertension, total cholesterol, and blood glucose) and serum 25 hydroxy vitamin D (25[OH]D) levels.

**Methods:**

Three hundred twenty-one participants were included in this study. Fasting blood tests were collected from all participants for biochemical measurements. Blood pressure and anthropometric measurements were also taken. A validated, semi-quantitative food frequency questionnaire was used to collect data on participants’ dietary intake. Dietary scores for the HFD pattern were calculated based on recommended food groups. MedDiet scores were calculated based on a previously validated method that contains 14 questions related to MedDiet. Both diet scores were classified into tertiles. Linear regression analyses were performed to assess the statistical significance of the tertile groups.

**Result:**

A significant association was found between HFD score and obesity when comparing the lowest tertile (27.3±4.6 kg/m^2^) of HFD scores with the medium tertile (29.2±5.7 kg/m2; *P* = 0.02). A higher HFD score was significantly associated with lower 25(OH)D levels (*P* = 0.02). In addition, a significant association was observed between MedDiet scores and 25(OH)D levels, with an increase in MedDiet score resulting in an increase in 25(OH)D levels (*P* = 0.01). Furthermore, a significant negative association between MedDiet scores and low-density lipoprotein levels was reported only in participants with CVD (*P* = 0.03).

**Conclusion:**

The results of this study revealed that HFD and MedDiet scores might have a role in the development of CVD and vitamin D deficiency among the Saudi Arabian population. Further studies are required using diet scores to assess the quality of dietary patterns and their association with an increased risk of diseases in Saudi Arabians.

## Popular scientific summary

- This is the first study to examine the association between dietary pattern scores and serum 25(OH)D levels among a sample of Saudi Arabian adults.
- A significant inverse association between high-fat dietary (HFD) pattern scores and serum 25(OH)D levels was reported, whereas greater adherence to MedDiet was associated with higher serum 25(OH)D levels.
- A significant positive association was found between HFD score and obesity.
- A significant negative association was found between MedDiet score and LDL levels in CVD participants.

The major cause of morbidity and mortality globally is cardiovascular disease (CVD), accounting for approximately 31% of all deaths (1). In Saudi Arabia, numerous studies have shown a high prevalence of CVD and associated risk factors (2–4). Unhealthy dietary patterns are the main risk factor for CVD, with almost 10,000,000 CVD deaths globally caused by dietary risk factors in 2017. The main dietary risk factors attributed to this number of deaths were a high sodium intake, low fruit intake, and low whole-grain intake (5). Evidence shows that reducing CVD risk depends on macronutrient replacement, that is, replacing saturated fat with unsaturated fat. Randomized controlled trials have confirmed that replacing saturated fatty acids with unsaturated fatty acids reduces CVD risk compared with refined carbohydrates (6–8). In addition, higher adherence to a healthy dietary pattern significantly reduced CVD risk by 0.78 in the general population, according to the findings of a meta-analysis based on longitudinal cohort studies (9).

Dietary pattern is the term used to describe a person’s food intake patterns over a period of time (10). Most nutrition research has examined a single nutrient or type of food in relation to a specific disease; however, the focus is currently on food-based approaches, such as dietary patterns. The study of dietary patterns is more effective because they mirror the real world, in which individuals consume entire meals and not isolated nutrients. Furthermore, eating behaviors cover a combination of various foods and a variety of nutrients that are more likely to interact with each other, and their shared effect may offer a comprehensive approach to preventing and treating numerous diseases (11, 12). One dietary pattern that has a favorable effect on the reduction of CVD risk is the Mediterranean diet (MedDiet). The MedDiet is characterized by a high intake of vegetables, fruits, nuts, seafood, and olive oil as sources of monounsaturated fatty acids, as well as low consumption of red meat and processed meat. Thus, the MedDiet is widely considered a healthy dietary pattern (13, 14). In a 10-year follow-up study, high adherence to the MedDiet, measured on a 10-point scale, caused a 0.85 and 0.88 reduction in CVD incidence and mortality, respectively (15). In the PREDIMED randomized primary prevention trial, adherence to the MedDiet plus extra-virgin olive oil and to the MedDiet plus nuts resulted in a reduction in atherosclerotic risk factors by 0.34 and 0.50, respectively, 4.8 years after follow-up (16).

A diet score is a tool used to assess the quality of a person’s dietary pattern according to recommendations or compliance with a defined dietary pattern. The score measures adherence to a combination of food groups and nutrient intake. It has also been used to assess adherence to dietary recommendations or healthy dietary patterns such as MedDiet (17, 18). Several studies have used the diet scoring approach to examine adherence to specific dietary patterns, as well as whether they are associated with an increased risk of diseases such as CVD (9, 19, 20).

Vitamin D deficiency is a serious problem in Saudi Arabia. National studies have reported serum 25(OH)D deficiency <50 mmol/L (<20 ng/mL) among various age groups in Saudi Arabia (21–23). In Saudi males, the prevalence of vitamin D deficiency reached 40%, whereas it reached 60% in Saudi females (24). Numerous scientific studies have steadily revealed an association between serum 25(OH)D deficiency and an increased risk of chronic diseases such as CVD (25, 26). To the best of our knowledge, this is the first study to examine the association between dietary pattern scores and serum 25(OH)D levels in Saudi Arabia. Therefore, the aim of this study is to examine the association of two diet scores, high-fat dietary (HFD) pattern scores and MedDiet scores, and CVD risk factors and serum 25 hydroxy vitamin D (25[OH]D) levels in a sample of Saudi Arabian adults, which may benefit planning programs that aim to prevent and reduce the risk of CVD and vitamin D deficiency in Saudi Arabia.

## Materials and methods

### Study population

The study design and population have been described in detail elsewhere (27). In brief, the study was conducted from May to October 2015 in the cities of Jeddah and Makkah, Saudi Arabia. The study sample included 325 participants (130 cases and 195 controls); however, only 321 participants completed the food frequency questionnaire (FFQ). Thus, 321 participants took part in this study: 129 participants with CVD (cases) and 192 participants without CVD (controls). CVD participants were recruited from the cardiology department in King Abdullah Medical City. The control group participants were recruited from family medicine clinics and nose and throat clinics, as well as ophthalmology clinics at Tunsi Private Hospital and King Abdulaziz University Hospital. All participants included in this study were male and female adults, either Saudis or residents in Saudi Arabia for at least 5 years. Participants with health conditions that affect vitamin D metabolism (e.g., kidney and liver diseases, hyperparathyroidism, and osteoporosis) were excluded. Eligible participants signed written informed consent forms before participating in the study. Ethical approval was sought from the Griffith University Human Research Ethics Committee (GU Ref No. MED/59/14/HREC), the Institutional Review Board at King Abdullah Medical City (IRB No. 15–194), and the Research Ethics Committee at King Abdulaziz University (Reference No. ll8–15).

### Data collection

Face-to-face interviewers were conducted with all participants to collect data regarding sociodemographic factors, including age, sex, marital status, level of education, and employment. In addition, data regarding family history of CVD and the use of vitamin D dietary supplements were collected. Height, weight, and blood pressure measurements were made using the hospital’s standard equipment after conducting the interviews. Body mass index (BMI) was calculated as weight (kilograms) divided by height (meters) squared. Obesity and overweight were defined according to the World Health Organization’s (WHO’s) definition. When the participants’ BMI was ≥30 kg/m², they were considered obese, and when the participants’ BMI was 25–29.9 kg/m², they were considered overweight (28). Hypertension was defined according to the WHO’s criteria as BP ≥140 mmHg for systolic blood pressure or ≥90 mmHg for diastolic blood pressure or both, as well as subjects on antihypertensive drugs (28). To measure total cholesterol, blood glucose, and serum levels of 25 hydroxy vitamin (25(OH)D), 10-mL blood samples were collected from all study participants through venipuncture. The serum samples were centrifuged at 2,000 rpm for 15 min. Thereafter, the blood samples were separated and kept frozen at −80°C for additional analyses. Participants with CVD had further blood tests, including low-density lipoprotein cholesterol (LDL), high-density lipoprotein cholesterol (HDL), and triglycerides. Serum 25(OH)D levels were assessed using a chemiluminescence microparticle immunoassay on the Architect system (Abbott). Vitamin D deficiency was defined as serum 25(OH)D concentrations of less than 10 ng/mL (<24.9 nmol/L), whereas vitamin D insufficiency was defined as serum 25(OH)D concentrations of 10–19.9 ng/mL (24.9–49.6 nmol/L). Adequate vitamin D serum levels were defined as serum concentrations of 20 ng/mL and above (≥49.6 nmol/L) (28). High total cholesterol was defined according to the Adult Treatment Panel III as total cholesterol ≥240 mg/dL, LDL cholesterol ≥160 mg/dL, HDL cholesterol **˂**40 mg/dL, and triglycerides ≥200 mg/dL or if the patients were on medications (28). Diabetes was defined according to the WHO’s criteria as fasting plasma glucose ≥ 126 mg/dL or as subjects being on medications for diabetes (28). A validated, semi-quantitative FFQ was used to collect data on intake of foods associated with an increased or decreased risk of CVD (29). The FFQ collects information related to the frequency of the intake of food groups, including cheese, red meat, variety meats (e.g., liver or kidneys), fresh or canned fish, delicatessen meat (including sausage), salted pies, pizzas, rolls or commercial sandwiches, French fries, cakes and pastries, nuts, butter, olive oil, fruits, and vegetables (29).

### HFD pattern questionnaire score

Dietary scores for HFD were calculated based on participants’ consumption of the recommended food groups. The frequency of consumption was scored on an ascending point scale, beginning with unhealthy items such as cheese, red meat, delicatessen meat (including sausage), pie and pizza, French fries, cakes, and butter at the bottom. The frequency of healthy food consumption (e.g., fruit, nuts, vegetables, and olive oil) was scored on a descending point scale (Supplementary Table 1).

**Table 1:** Participants sociodemographic characteristics according to high-fat dietary (HFD) pattern score

	Low HFD score (*n* = 81)	Medium HFD score (*n* = 124)	High HFD score (*n* = 116)	*P*
Sex M F	54 (66.7) 27 (33.3)	80 (64.5) 44 (35.5)	69 (59.5) 47 (40.5)	0.54
Age group (years) 20–29 30–39 40–49 50–59 60–65 >65	1 (1.2) 10 (12.3) 19 (23.5) 21 (25.9) 22 (27.2) 8 (9.9)	1 (0.8) 7 (5.6) 22 (17.7) 47 (37.9) 22 (17.7) 25 (20.2)	3 (2.6) 9 (7.8) 19 (16.4) 50 (43.1) 18 (15.5) 17 (14.7)	0.09
Marital status Single Married Divorced Widowed	5 (6.2) 65 (80.2) 6 (7.4) 5 (6.2)	14 (11.3) 93 (75) 8 (6.5) 9 (7.3)	22 (19) 69 (59.5) 14 (12.1) 11 (9.5)	0.04
Family history No Yes	60 (74.1) 21 (25.9)	69 (55.6) 55 (44.4)	61 (52.6) 55 (47.4)	0.006
Education No formal education Up to primary level Secondary school High school Bachelor degree Master degree	21 (25.9) 13 (16) 9 (11.1) 13 (16) 22 (27.2) 3 (3.7)	29 (23.4) 14 (11.3) 18 (14.5) 16 (12.9) 44 (35.5) 3 (2.4)	10 (8.6) 8 (6.9) 14 (12.1) 28 (24.1) 50 (43.1) 6 (5.2)	0.01
Employment Employed Unemployed Student Self employed Retired House wife	46 (56.8) 13 (16) 0 5 (6.2) 10 (12.3) 7 (8.6)	67 (54) 16 (12.9) 0 6 (4.8) 23 (18.5) 12 (9.7)	70 (60.3) 14 (12.1) 2 (1.7) 4 (3.4) 19 (16.4) 7 (6)	0.68

Values represented are frequencies.

Chi-square test was used to test for significant differences.

*P* < 0.05 was considered statistically significant.

### MedDiet pattern questionnaire score

The MedDiet score was calculated based on a previously validated method (18). The questionnaire contains 14 questions related to MedDiet; however, this study’s questionnaire contained only nine questions out of the 14. The nine questions related to the use of olive oil and amount, as well as the consumption of fruits, vegetables, butter, nuts, sweets and pastries, sausage or processed meat, red meat, and fish (Supplementary Table 2). The validated FFQ used in this study did not contain questions related to poultry, legumes, tomato sauce, carbonated drinks, and wine; thus, the score was out of nine instead of 14.

**Table 2:** Association between cardiovascular diseases (CVD) risk factors of study participants and high-fat dietary (HFD) pattern score

	Low HFD score (*n* = 81)	Medium HFD score (*n* = 124)	High HFD score (*n* = 116)	*P*
Body mass index (BMI) (kg/m^2^)	27.3 ± 4.6	29.2 ± 5.7	27.7 ± 4.7	0.02
Blood glucose (mmol/L)	6.5 ± 2.1	7.4 ± 5.0	6.7 ± 4.1	0.64
Total cholesterol (mmol/L)	4.7 ± 1.4	4.3 ± 1.1	4.7 ± 1.2	0.11
Systolic blood pressure (mmHg)	122.5 ± 18	124.36 ± 18.8	120.24 ± 17.0	0.28
Diastolic blood pressure (mmHg)	73.9 ± 12.0	71.8 ± 12.5	73.2 ± 11.1	0.56
25 hydroxy vitamin D (ng/ml)	18.8 ± 8.4	19.1 ± 10.1	17.9 ± 8.8	0.02

Values are mean± SD.

Linear regression was used to test for significant differences, and estimates were adjusted for age, sex, CVD, family history of CVD, vitamin D supplements, sun exposure time, the use of sunscreen, BMI, and total cholesterol.

*P* < 0.05 was considered statistically significant.

### Statistical analysis

Statistical analyses were performed using SPSS Version 26. Data presented in tables show the mean ± SD for continuous variables and frequency for categorical variables. Chi-square tests were used to examine differences between categorical variables. A normal distribution was tested for all continuous variables, and log-transformation data were used for the glucose value because it skewed normality. Participants’ responses to the HFD pattern and MedDiet questionnaires were classified into tertiles depending on their diet scores. Participants’ HFD scores ranged from 6 to 33 points; thus, groups were divided into low-risk (6–13, *n* = 81), medium-risk (14–18, *n* = 124), and high-risk (19–33, *n* = 116) groups based on the SPSS analysis of tertiles. MedDiet scores were divided into low adherence (0–3, *n* = 80), medium adherence (4–5, *n* = 149), and high adherence (6–9, *n* = 92), similar to a previous study (30). Linear regression analyses were performed to assess the statistical significance of the tertile groups. Due to the small sample size, patient and control groups were combined for analysis. Estimates were adjusted for confounding factors such as age, sex, CVD, family history of CVD, vitamin D supplements, sun exposure time, the use of sunscreen, BMI, and total cholesterol. A *p*-value of <0.05 was considered statistically significant.

## Results

### HFD pattern

The sociodemographic characteristics (e.g., age, sex, educational level, marital status, family history of CVD, and employment) of the study participants are presented in Table 1, stratified by HFD scores. Among the tertile groups for HFD score, the percentages of males and females were 16.8 and 8.4% in the low-risk tertile, 24.9 and 13.7% in the medium-risk tertile, and 21.4 and 14.6% in the high-risk tertile, respectively. The percentages of married participants were significantly higher: 28.9% in the medium-risk tertile and 21.4% in the high-risk tertile compared to 20.2% in the low-risk tertile (*P* = 0.04). The more educated participants (i.e., university graduates with bachelor’s degrees or higher) represented 13.7% in the medium-risk tertile and 15.5% in the high-risk group compared to 6.8% in the low-risk group.

**Fig. 1:**
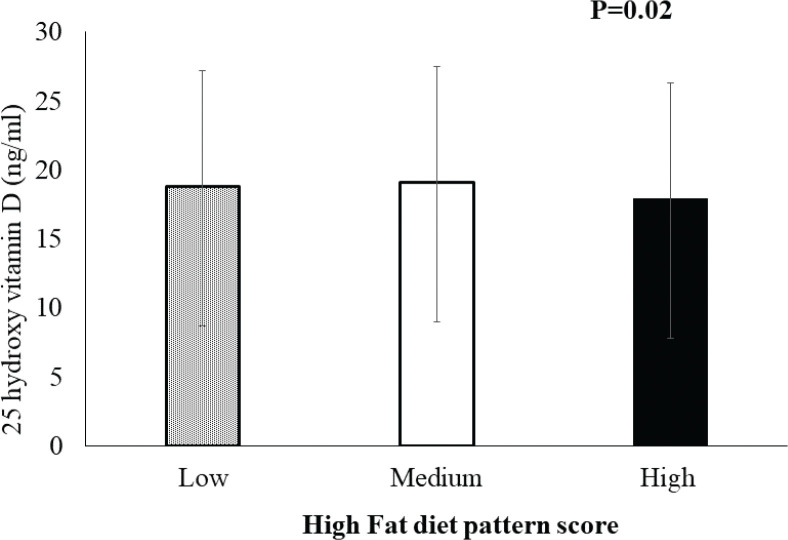
A significant association between high-fat dietary (HFD) pattern score and 25 hydroxy vitamin D levels, with an increase in high-fat dietary (HFD) pattern score resulting in a decrease in 25 hydroxy vitamin D levels (*P* = 0.02).

A significant negative association between HFD score and serum 25(OH)D levels was seen after adjustment for age, sex, CVD, family history of CVD, vitamin D supplements, sun exposure time, the use of sunscreen, BMI, and total cholesterol (Fig. 1) when comparing the high-risk tertile [17.9 ± 8.8 ng/mL (44.68 nmol/L)] of HFD scores to the low- and medium-risk tertiles [18.8 ± 8.4 ng/mL (46.92 nmol/L) and 19.1 ± 10.1 ng/mL (47.67 nmol/L), respectively; *P* = 0.02]. Individuals in the high-risk tertile had a 2.2 times increased risk of vitamin D deficiency compared to the low-risk tertile.

Furthermore, a significant positive association was observed between the HFD score and obesity after adjustment for age, sex, CVD, family history of CVD, vitamin D supplements, sun exposure time, the use of sunscreen, BMI, and total cholesterol when comparing the low-risk tertile (27.3±4.6 kg/m^2^) of HFD scores with the medium-risk tertile (29.2±5.7 kg/m^2^; *P* = 0.02). Individuals in the medium-risk tertile had a 1.7 times higher risk of obesity than those in the low-risk tertile (Table 2). However, no significant differences existed between the low and high tertiles of HFD scores and obesity. No significant associations between the HFD score and total cholesterol, glucose levels, or blood pressure were observed.

### MedDiet pattern

The sociodemographic characteristics (e.g., age, sex, educational level, marital status, family history of CVD, and employment) of the study participants are presented in Table 3, stratified by MedDiet scores. Among tertile groups for MedDiet score, the percentages of males and females were 68.8 and 31.3% in the low MedDiet score tertile, 63.8 and 36.2% in the medium MedDiet score tertile, and 57.6 and 42.4% in the high MedDiet score tertile, respectively. The percentages of married participants were significantly higher in the medium MedDiet score tertile (78.5%) and in the high MedDiet score tertile (68.5%) than in the low MedDiet score tertile (58.8%) (*P* = 0.04). The percentages of educated participants (i.e., university graduates with bachelor’s degrees or higher) were significantly higher in the high MedDiet score tertile (44.6%) than in the medium MedDiet score tertile (26.8%) and the low MedDiet score tertile (43.8%) (*P* = 0.01).

**Table 3:** Participants’ sociodemographic characteristics according to the Mediterranean diet (MedDiet) score

	Low MedDiet score (*n* = 80)	Medium MedDiet score (*n* = 149)	High MedDiet score (*n* = 92)	*P*
Sex M F	55 (68.8) 25 (31.3)	95 (63.8) 54 (36.2)	53 (57.6) 39 (42.4)	0.31
Age group (years) 20–29 30–39 40–49 50–59 60–65 >65	3 (3.8) 5 (6.3) 13 (16.3) 28 (35) 15 (18.8) 16 (20)	1 (0.7) 14 (9.4) 29 (19.5) 58 (38.9) 24 (16.1) 23 (15.4)	1 (1.1) 7 (7.6) 18 (19.6) 32 (34.8) 23 (25) 11 (12)	0.60
Marital status Single Married Divorced Widowed	14 (17.5) 47 (58.8) 8 (10) 11 (13.8)	16 (10.7) 117 (78.5) 10 (6.7) 6 (4)	11 (12) 63 (68.5) 10 (10.9) 8 (8.7)	0.04
Family history No Yes	36 (45) 44 (55)	87 (58.4) 62 (41.6)	67 (72.8) 25 (27.2)	0.001
Education No formal education Up to primary level Secondary school High school Bachelor degree Master degree	7 (8.8) 6 (7.5) 11 (13.8) 18 (22.5) 35 (43.8) 3 (3.8)	33 (22.1) 19 (12.8) 24 (16.1) 28 (18.8) 40 (26.8) 5 (3.4)	20 (21.7) 10 (10.9) 6 (6.5) 11 (12) 41 (44.6) 4 (4.3)	0.01
Employment Employed Unemployed Student Self-employed Retired House wife	48 (60) 7 (8.8) 2 (2.5) 3 (3.8) 16 (20) 4 (5)	77 (51.7) 22 (14.8) 0 8 (5.4) 26 (17.4) 16 (10.7)	58 (63) 14 (15.2) 0 4 (4.3) 10 (10.9) 6 (6.5)	0.18

Values represented are frequencies.

Chi-square test was used to test for significant differences.

*P* < 0.05 was considered statistically significant.

**Fig. 2:**
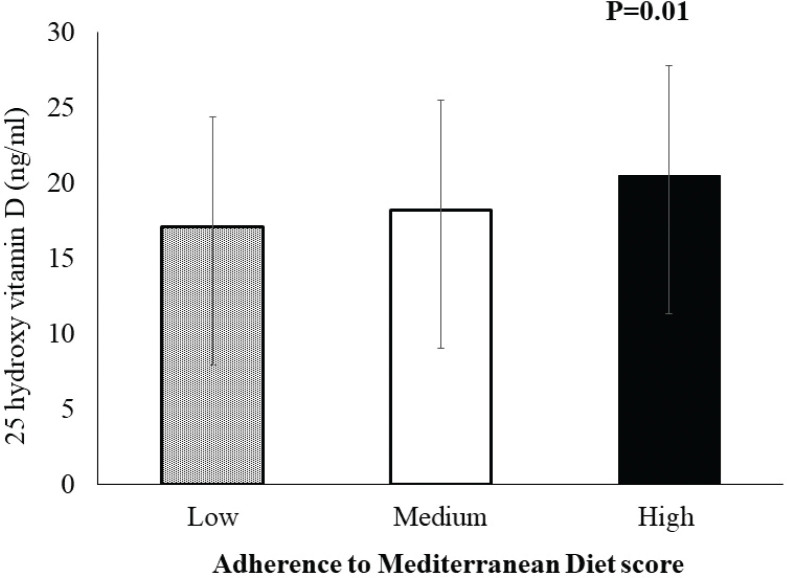
A significant association between Mediterranean Diet (MedDiet) score and 25 hydroxy vitamin D levels, with an increase in Mediterranean Diet (MedDiet) score resulting in an increase in 25 hydroxy vitamin D levels (*P* = 0.01).

A significant association between MedDiet score and serum 25(OH)D levels was observed after adjustment for age, sex, CVD, family history of CVD, vitamin D supplements, sun exposure time, the use of sunscreen, BMI, and total cholesterol, with the increase in MedDiet score resulting in an increase in serum 25(OH)D levels (*P* = 0.01; Fig. 2).

**Fig. 3:**
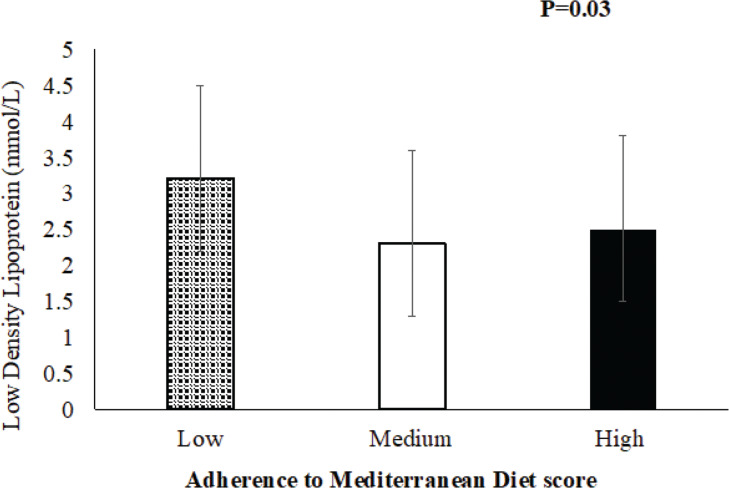
A significant association between adherence to Mediterranean Diet (MedDiet) score and low-density lipoprotein (LDL) levels in CVD participants (*P* = 0.03), where high or medium adherence to Mediterranean Diet (MedDiet) was associated with a decrease in the level of LDL compared to the low adherence group.

The results for the association between CVD risk factors and MedDiet score are shown in Table 4. No significant associations between MedDiet score and obesity, total cholesterol, glucose levels, or blood pressure were observed. A protective effect was seen between MedDiet score and LDL levels in participants with CVD only (*P* = 0.03), suggesting that high or medium adherence to the MedDiet (2.5 ± 1.0 and 2.3 ± 0.8 mmol/L, respectively) was associated with a decreased level of LDL compared to low adherence (3.2 ± 1.3 mmol/L) in participants with CVD (Fig. 3). No further significant associations between HFD score and MedDiet score with other lipid profile levels of CVD participants were observed (Supplementary Table 3).

**Table 4:** Association between cardiovascular diseases (CVD) risk factors of study participants and Mediterranean Diet (MedDiet) score

	Low MedDiet score (*n* = 80)	Medium MedDiet score (*n* = 149)	High MedDiet score (*n* = 92)	Model 2 *P*
Body mass index (BMI) (kg/m^2^)	28.1 ± 4.4	28.2 ± 5.6	28.3 ± 5.09	0.64
Blood glucose (mmol/L)	6.7 ± 4.1	7.0 ± 3.5	7.2 ± 4.8	0.47
Total cholesterol (mmol/L)	4.6 ± 1.2	4.4 ± 1.2	4.7 ± 1.3	0.61
Systolic blood pressure (mmHg)	120.6 ± 16.1	123.4 ± 19.6	122.2 ± 16.8	0.32
Diastolic blood pressure (mmHg)	73.1 ± 11.4	73.7 ± 12.0	71.2 ± 11.9	0.05
25 hydroxy vitamin D (ng/ml)	17.1 ± 7.3	18.2 ± 9.9	20.5 ± 9.2	0.01

Values are mean± SD.

Linear regression was used to test for significant differences, and estimates were adjusted for age, sex, CVD, family history of CVD, vitamin D supplements, sun exposure time, the use of sunscreen, BMI, and total cholesterol.

*P* < 0.05 was considered statistically significant.

## Discussion

This study attempted to investigate whether HFD scores and MedDiet scores were associated with CVD risk factors, including serum glucose, obesity, total cholesterol, blood pressure, and serum 25(OH)D levels, among population of Saudi Arabia. Findings from this case-control study showed a significant positive association between HFD score and obesity when comparing the low-risk tertile of HFD scores with the medium-risk tertile but not for the high-risk tertile. In addition, the results indicated an inverse association between HFD score and serum 25(OH)D levels, whereas greater adherence to MedDiet was associated with higher serum 25(OH)D levels. In CVD participants, adherence to MedDiet resulted in decreased LDL levels, which may suggest a protective effect of MedDiet among participants with CVD.

A previous cross-sectional study conducted on children and adolescents (2–19 years) from the United States observed an association between dietary pattern scores and 25(OH)D levels (31). The study used two types of diet scoring. The first was a high-fat-low-vegetable dietary pattern based on the consumption of condiments, mixed dishes, snacks and sweets, refined grains, pizza, meats, processed meats, poultry, fish and other seafood, high-fat dairy, coffee or tea, and starchy vegetables. The second was a prudent dietary pattern based on the consumption of vegetable groups, fruits, mixed dishes, other fats, fish and other seafood, and meats. The study reported an association between both diet scores and the 25(OH)D concentration in the unadjusted model but not in the adjusted model. The results suggest that children and adolescents with high adherence to the high-fat-low-vegetable dietary pattern had lower serum 25(OH)D concentrations than those with low adherence (31). However, this study examined different age groups and participants with different health statuses, as well as slightly different components of the dietary patterns included in the scores compared to this study.

Sunlight exposure is a well-known major source of serum 25(OH)D (32). However, studies have demonstrated that the consumption of certain vitamin D-rich foods, including oily fish and egg yolks, had a significant positive effect on serum 25(OH)D levels. In addition, exposure to sunlight can be influenced by many factors, including skin color, age, and the use of sunscreen (33). In addition, national studies conducted in Saudi Arabia have indicated that people have negative attitudes toward sun exposure. Individuals face several barriers to sun exposure, such as hot weather and wearing clothes covering most of the body (34). In fact, in this study, information regarding sun exposure time and the use of sunscreen was entered in the regression model as confounding factors to identify the association between dietary pattern scores and serum 25(OH)D levels. Hence, dietary patterns can be an essential contributor to serum 25(OH)D. In addition, to our knowledge, this is the first study to examine the association between dietary pattern scores and serum 25(OH)D levels among a Saudi Arabian population.

Regarding the association between HFD scores and obesity, two cross-sectional studies of Mexican adolescents and European adults confirmed that being in the highest tertile of a high-protein–high-fat pattern and a Western pattern was associated with a higher BMI compared to the highest tertile of people following a prudent dietary pattern (35, 36). However, in this study, significance was only found between participants in the medium and low HFD tertiles. The reason might be the small sample size and the sample’s health status, but it could also be that individuals at higher risk may be physically active or their consumption from energy was low.

Regarding the association between MedDiet and serum 25(OH)D levels, a cross-sectional study of 92 healthy obese children and children with metabolic syndrome from Croatia showed that adherence to MedDiet was not associated with 25(OH)D concentrations (37). Furthermore, evidence indicates that vitamin D deficiency is prevalent in Mediterranean countries where the population highly adheres to MedDiet. Approximately 35–40% of the population in Croatia, Italy, and Spain had serum 25(OH)D concentrations mmol/L (<20 ng/mL) (38). In comparison, this study’s findings indicated that high or medium adherence to a MedDiet was associated with a high level of serum 25(OH)D compared to low adherence. The difference in results might be due to the different cut-off values being used to define vitamin D deficiency worldwide.

Similar to our findings, a cross-sectional study conducted among 1,213 Flemish adults of both sexes reported that MedDiet score was negatively associated with LDL levels among male participants (30). Moreover, findings from a meta-analysis that included six intervention studies on MedDiet (with a total sample size of 3,227 participants) showed that dietary intervention with MedDiet resulted in a significant reduction in LDL levels by −0.07 mmol/L (39). Nevertheless, another meta-analysis including six interventions does not observe a reduction in LDL with the MedDiet; however, the MedDiet affected other CVD risk factors including BMI, blood pressure, total cholesterol, and blood glucose (40). The abundance of unsaturated fatty acids, polyphenols, and antioxidant content in the MedDiet may be a possible explanation for its protective effect against CVD risk factors. These nutrients play different crucial roles, including reducing oxidative stress, promoting anti-inflammation, and improving parameters of endothelial function (41–43).

This study has some limitations. The study design is cross-sectional and cannot prove causality compared to a prospective design. Additionally, our sample size was relatively small. Self-reporting bias by participants is increased when using the FFQ. The study participants included controls and cases combined to form one group; however, the results were adjusted to include CVD in all the analyses. Lipid profiles, such as triglycerides, HDL, and LDL, were only available for the CVD participants, which influenced the observed results. In the MedDiet score, some components of diet were missing in our FFQ, such as legume and tomato consumption (separate from the vegetable question). On the contrary, the main strength of this study is that two diet scores were used, and both showed associations with serum 25(OH)D levels in the Saudi Arabian population, making this the first study to examine this issue in Saudi Arabia.

## Conclusions

In conclusion, the HFD score was significantly associated with obesity when comparing the lowest tertile with the medium tertile but not with the highest tertile. In addition, a high HFD score was significantly associated with decreased serum 25(OH)D levels, while a high MedDiet score was significantly associated with increased serum 25(OH)D levels. In CVD participants, greater adherence to the MedDiet was associated with decreased LDL levels as an indicator of CVD risk. These results suggest that compliance with a healthy dietary pattern rich in fruits, vegetables, fish, olive oil, and poultry and with reduced saturated fatty acids, red meat, and processed meat has beneficial effects on general health. In addition, based on our results, dietary patterns might have a role in the development of CVD as well as vitamin D deficiency in Saudi Arabia. Further studies should use diet scores to assess the quality of dietary patterns and their association with an increased risk of diseases in Saudi Arabia.

## Ethics approval

The study was conducted according to the Griffith University Human Research Ethics Committee (GU Ref No: MED/59/14/HREC), the Institutional Review Board at King Abdullah Medical City (IRB No: 15–194), and the Research Ethics Committee at King Abdulaziz University (Reference No ll8–15).

## Authors’ contribution

NMA collected the data and involved in the original study. IMS performed the statistical analysis. IMS and NMA wrote the first draft of the manuscript. NMA reviewed the manuscript and had the primary responsibility for the final content. All authors read and approved the final manuscript.
